# Vibrational stability improvement of a mirror system using active mass damping

**DOI:** 10.1107/S1600577524006490

**Published:** 2024-08-08

**Authors:** Shijing He, Haoran Yuan, Tianyu Wu, Nuo Chen, Xinyu Zhang, Zhizhuo Wang, Xuerong Liu, Fang Liu

**Affiliations:** a Center for Transformative Science ShanghaiTech University 393 Huaxia Middle Road Shanghai201210 People’s Republic of China; University of Essex, United Kingdom

**Keywords:** active vibration control, active mass damper, spillover effect, free-electron laser

## Abstract

Addressing the demand for high stability of beamline instruments at the SHINE facility, a high stability mirror regulating mechanism has been developed for mirror adjustments. Active mass damping was adopted to attenuate pitch angle vibrations of mirrors caused by structural vibrations. An internal absolute velocity feedback was used to reduce the negative impact of spillover effects and to improve performance. The experiment was conducted on a prototype structure of a mirror regulating mechanism, and results showed that the vibration RMS of the pitch angle was effectively attenuated from 47 nrad to 27 nrad above 1 Hz.

## Abstract 2

Active damping with internal absolute velocity feedback was implemented to damp the angular vibration of a mirror holder for free-electron laser beam transportation.

## 1. Introduction

Advanced X-ray light sources have become very important tools for research in modern science applications due to their high brightness, short pulse and good coherence. Compared with traditional synchrotron radiation sources, free-electron lasers (FELs) are characterized by higher peak brightness, shorter pulse width and better coherence (Pellegrini & Stöhr, 2003 ▸; Zhao & Feng, 2018 ▸), and are hence considered to be a new generation of light sources (Georgescu, 2020 ▸). As part of the Zhangjiang scientific facility group of advanced light sources, Shanghai High repetitioN rate XFEL and Extreme light facility (SHINE) is expected to achieve ultra-high peak brightness and average brightness, high repetition rate and femtosecond-level ultrafast pulses, as well as nanoscale ultra-high spatial resolution and femtosecond level ultrafast time resolution.

X-ray beam transportation is up to 1 km long, in order to take full advantage of the surface smoothness of the beamline optical devices (Siewert *et al.*, 2019 ▸), as well as the pointing stability – the angular demands of mirrors in the transportation should be less than 50 nrad. For such high requirements, disturbance from the tunnel floor, motion devices and cooling devices (Li *et al.*, 2011 ▸) cannot be ignored. Most of the disturbances will be attributed to structural vibrations of mechanical structures which will then affect the beam transportation stability (Houghton *et al.*, 2021 ▸).

In order to improve the stability of instruments at advanced light sources, passive vibration isolation (Mangra *et al.*, 1996 ▸; Van Vaerenbergh *et al.*, 2008 ▸) is generally used in engineering: the influence of vibration on the instrument is improved by optimizing the physical design of the mechanical structure. However, passive vibration isolation is a poor suppression effect for low-frequency vibrations. Furthermore, it is difficult to increase damping in a vacuum, and damping materials age quickly (Liu *et al.*, 2020 ▸).

With the development of microelectronics technology, research is increasingly focused on the active vibration reduction method, which can effectively damp structural resonances and low-frequency vibrations (Guoping *et al.*, 2004 ▸), with good flexibility. For active vibration control, the actuator arrangement of active vibration isolation is often limited by the actual mechanical structure, while the actuator position of active mass damping (AMD) (Gonzalez Diaz, 2007 ▸) is independent. However, AMD could cause serious resonance at its natural frequency (required to be lower than the controlled band), called the ‘spillover effect’, so the problem caused by the ‘spillover effect’ is intolerable for all-frequency sensitive applications.

In this work, an internal absolute velocity feedback (IAVF) is proposed for AMD to reduce the influence of the spillover effect, and to improve the modal vibration damping performance of the controlled structure. The angular pitch vibration attenuation performance was demonstrated on a mirror-adjusting mechanism for the SHINE project.

## 2. Model of active mass damping

**Figure 1:**
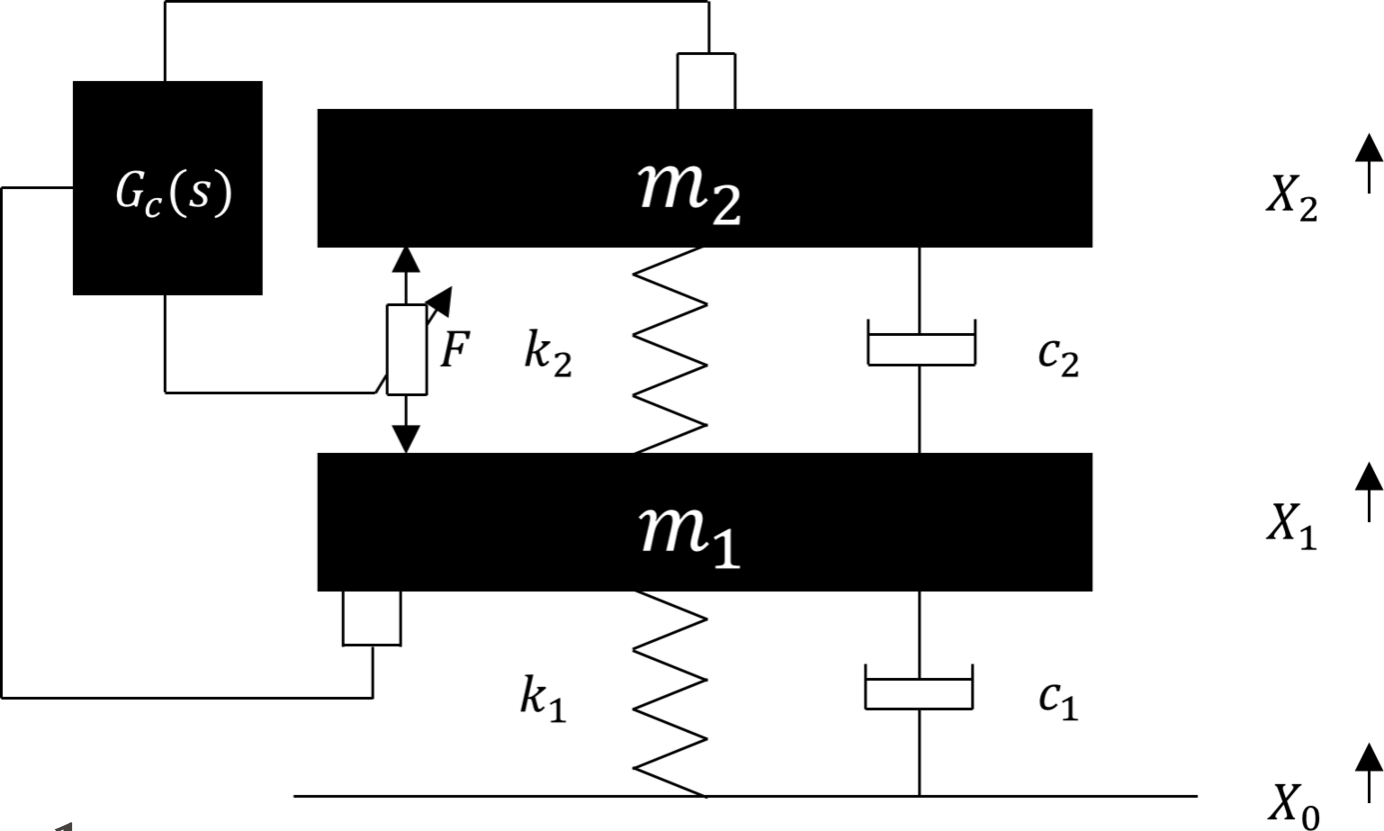
Two-degrees-of-freedom model of the active mass damping system.

**Figure 2:**
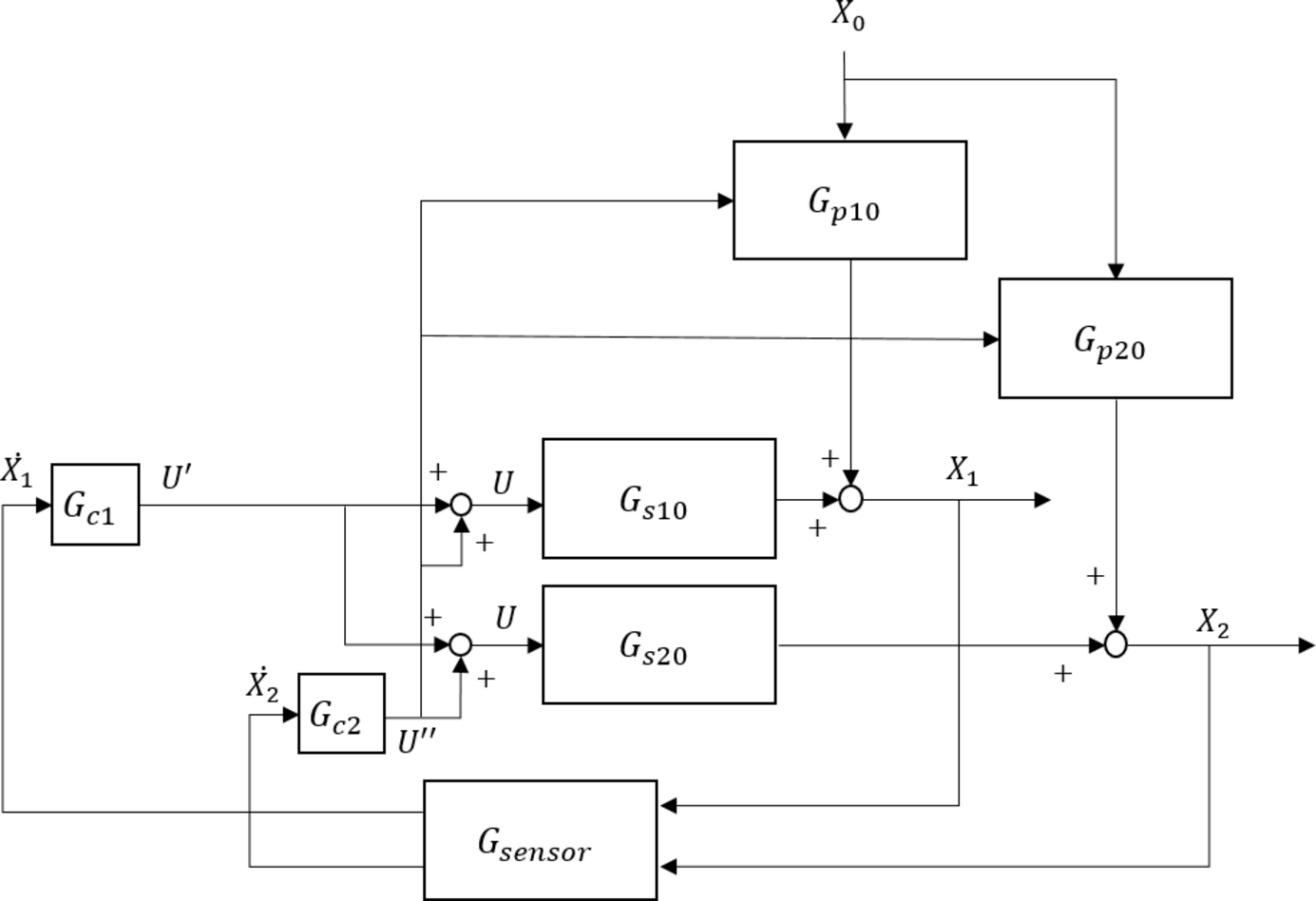
Control diagram of AMD with IAVF.

To attenuate structural vibrations, AMD is introduced. For simplicity, a two-degrees-of-freedom model is built. The principle of AMD is shown in Fig. 1 ▸, where *m*_1_ is the mass of the controlled structure and *m*_2_ is the mass of the active mass damper (AMDer). The dynamic equation of the system is
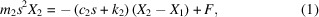

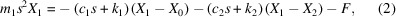
where *X*_1_(*s*), *c*_1_ and *k*_1_ are the Laplace transform of the absolute displacement, damping coefficient and stiffness of the controlled structure, respectively; *X*_2_(*s*), *c*_2_ and *k*_2_ are the Laplace transform of the displacement, damping coefficient and stiffness of the AMDer, respectively. *F* is the secondary force generated by a voice coil motor (VCM), expressed as
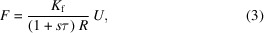
where *K*_f_, *R* and τ are the force constant, coil resistance and electrical time constant of the VCM, respectively (the back electromotive force was ignored here). *U* is the driving voltage of the VCM computed by the feedback controller, which, in the ordinary AMD, is described as
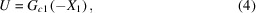
and the PID control algorithm *G*_c1_ is simply expressed as
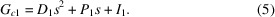
To reduce the spillover effect and improve the modal vibration attenuation performance, an IAVF is introduced to increase the active damping of the AMDer. A control diagram is illustrated in Fig. 2 ▸, according to which the driving voltage of the VCM became
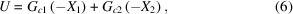
where
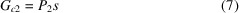
is part of the control algorithm for IAVF. According to all formulas above, with
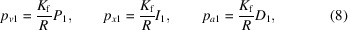

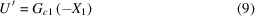
and
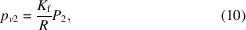
the vibrational transfer function from ground to controlled object, *G*_*p*1_, and that from *U*′ to controlled object vibration, *G*_*s*1_, are expressed as
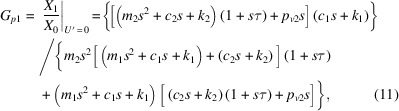

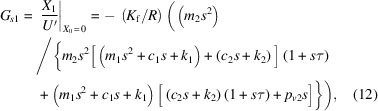
and the vibrational transfer function from the ground to the internal mass of the AMDer, *G*_*p*1_, and that from *U*′ to the internal mass of the AMDer, *G*_*s*1_, are expressed as
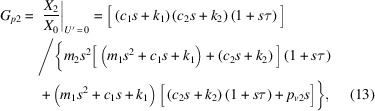

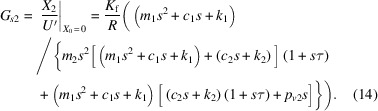
In particular, the definitions of *G*_*p*10_, *G*_*s*10_, *G*_*p*20_, *G*_*s*20_ in Fig. 2 ▸ are, respectively,
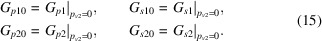
Considering the closed-loop transfer function by Fig. 2 ▸ (ignoring the dynamic characteristic of sensor),
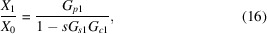
the control performance
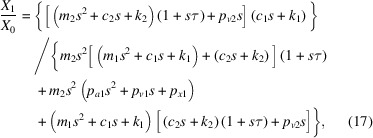
reflects the transmissibility from the ground to the controlled structure with AMD. In this work, velocity feedback was mainly adopted, since it is effective for multi-mode attenuation.

**Figure 3:**
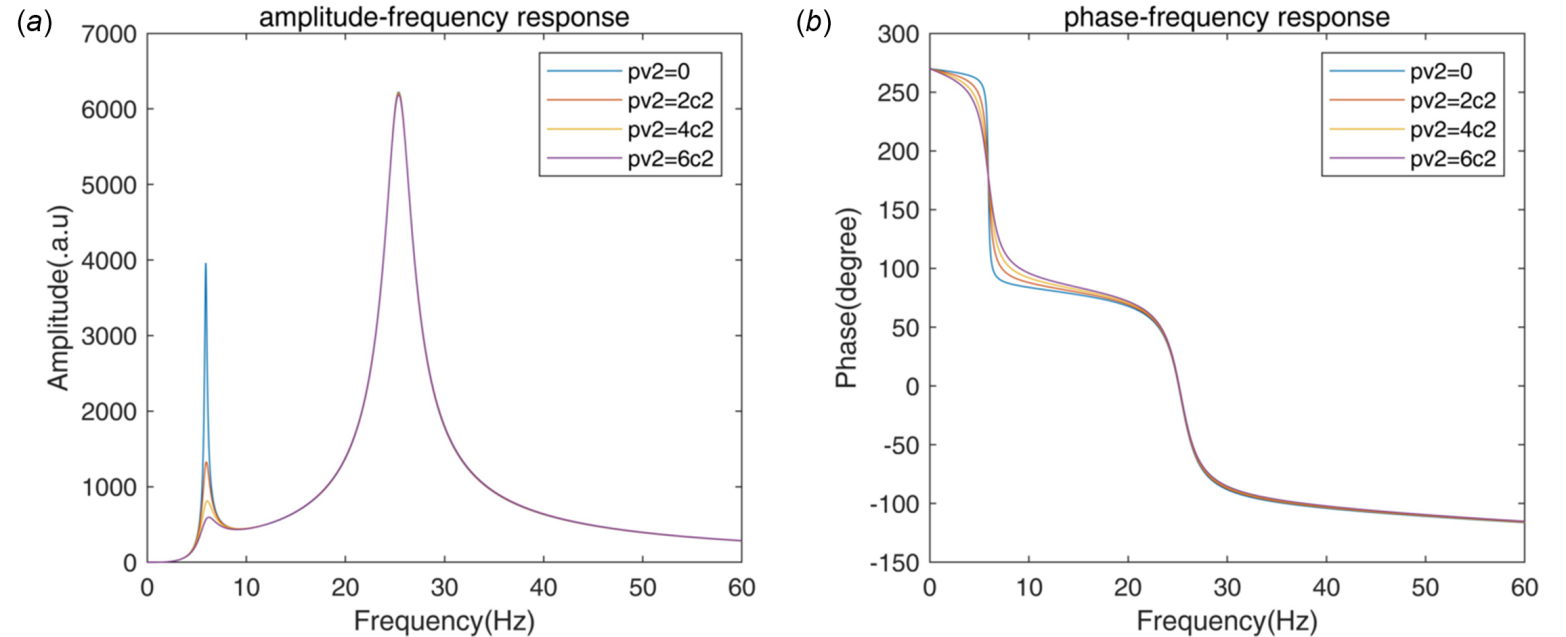
Open-loop frequency response of unity gain exterior velocity feedback.

According to formula (12), the open-loop frequency response (OLFR) of the unit-gain velocity feedback, 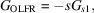
with the parameters listed in Table 1 ▸ is shown in Fig. 3 ▸(*a*). There are two resonant peaks in the amplitude–frequency response curve: one with lower natural frequency and lower amplitude, which is caused by the AMDer, the other with higher natural frequency and higher amplitude, which is from the mode of the controlled structure. For the phase–frequency response curve, the phase starts from 270° at 0 Hz, shows lag from about 250° to about 90° around the natural frequency of resonance of the AMDer, from about 70° to about −100° is around the natural frequency of the resonance of the controlled structure, and continues the lag slowly as the frequency increases. The crossing at 180° of the phase near the natural frequency of the resonance of the AMDer implies the spillover effect: the vibration of the closed-loop system at this frequency will be amplified when the gain increases, and divergent when the gain exceeds the margin. The influence of IAVF gain, *p*_*v*2_, is considered: as *p*_*v*2_ increases, the peak values of the open-loop amplitude–frequency response at the natural frequency of the AMD, |*G*_OLFR_(*j*ω_*a*_)|, drops visibly, while the amplitude at the *k*th natural frequency of the controlled structure, |*G*_OLFR_(*j*ω_*k*_)|, is almost invariant. According to
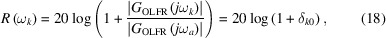
the stability–performance formula proposed by Gonzalez Diaz (2007 ▸), where 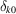 = 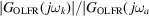, the performance of modal vibration attenuation could be improved by IAVF.

**Figure 4:**
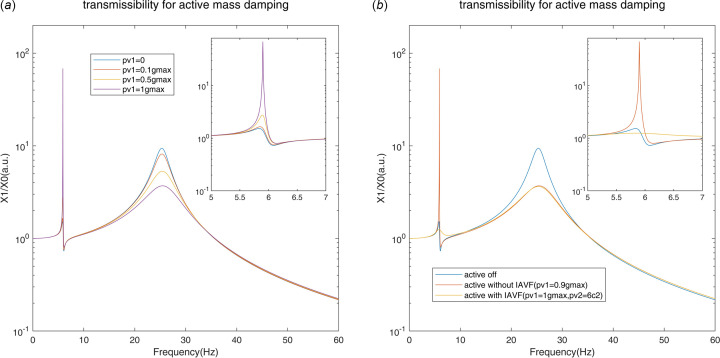
Transmissibility for AMD without IAVF (*a*) and with IAVF (*b*).

According to formula (17), the transmissibility of AMD is shown in Fig. 4 ▸. The active damping effect at the natural frequency of the controlled structure is significantly improved as the gain [*g*_max_ = 1/|*G*_OLFR_(*j*ω_*a*_)|] is raised; however, the spillover effect at the natural frequency of the AMDer increases. With the IAVF, it is obvious that the increase at AMD resonance is suppressed, while the same vibration attenuation performance is achieved at the resonance of the controlled structure. As a cost, the frequency range of the spillover effect is widened due to the active damping effect of IAVF.

## 3. Introduction of a test setup

To test the performance of the AMDer with IAVF, a prototype of the AMDer was built, installed and tested at the mirror regulating mechanism for the SHINE offset mirror.

### 3.1. Introduction of the mirror regulating system

**Figure 5:**
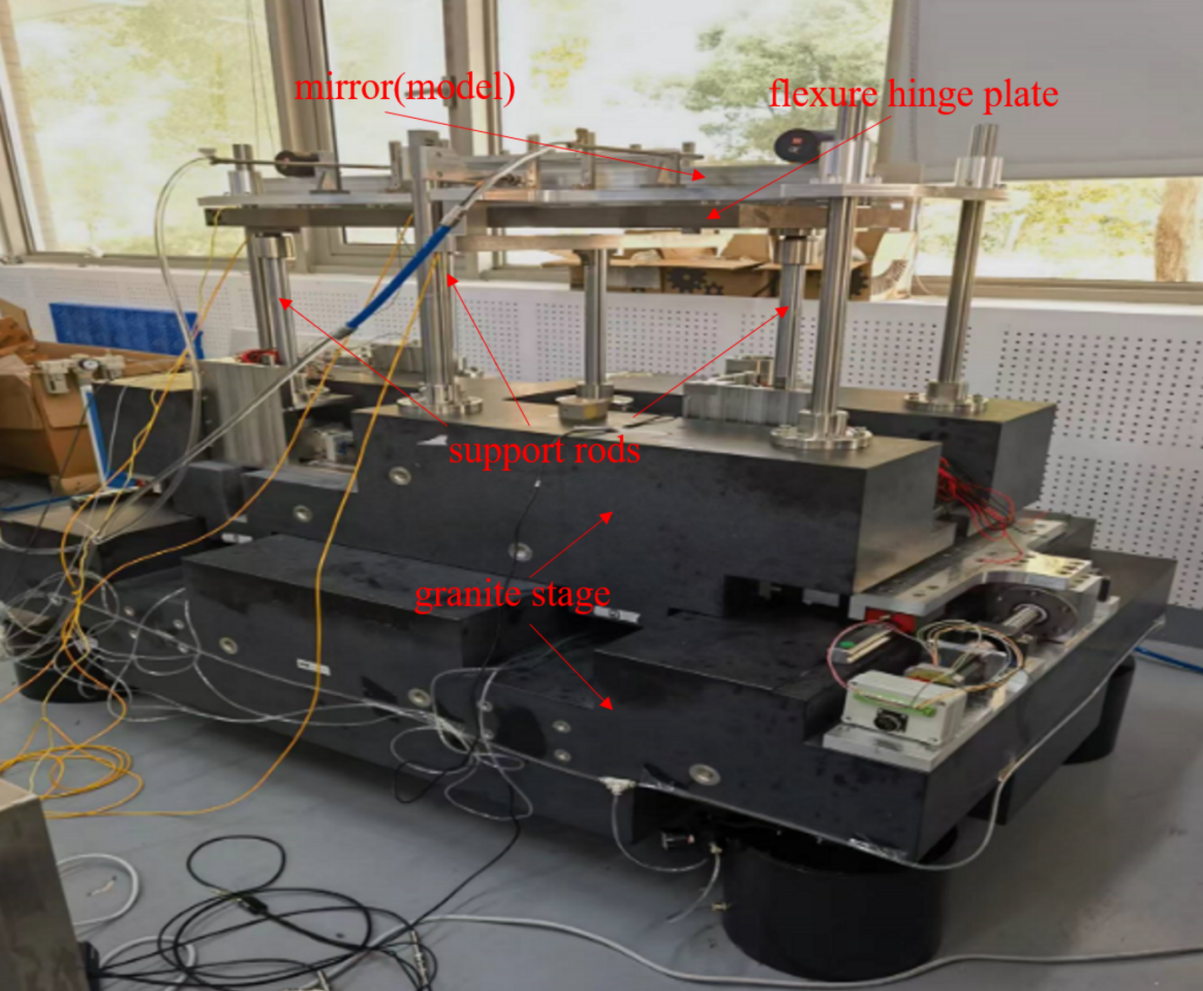
The prototype of the offset mirror adjustment system for the SHINE beamline.

The whole adjusting mechanism was designed as shown in Fig. 5 ▸. A detailed description has been given by Liu *et al.* (2023 ▸). A double-layer granite base was used for coarse pitch angle adjustment and horizontal translation, and the mirror holder was supported by three vertical adjustments to meet the vertical, roll and yaw adjustments. Since the pitch angle is the most critical angle that we need to deflect or focus the beam, a fine pitch adjustment was designed by flexures on the mirror holder driven by a piezo actuator.

**Figure 6:**
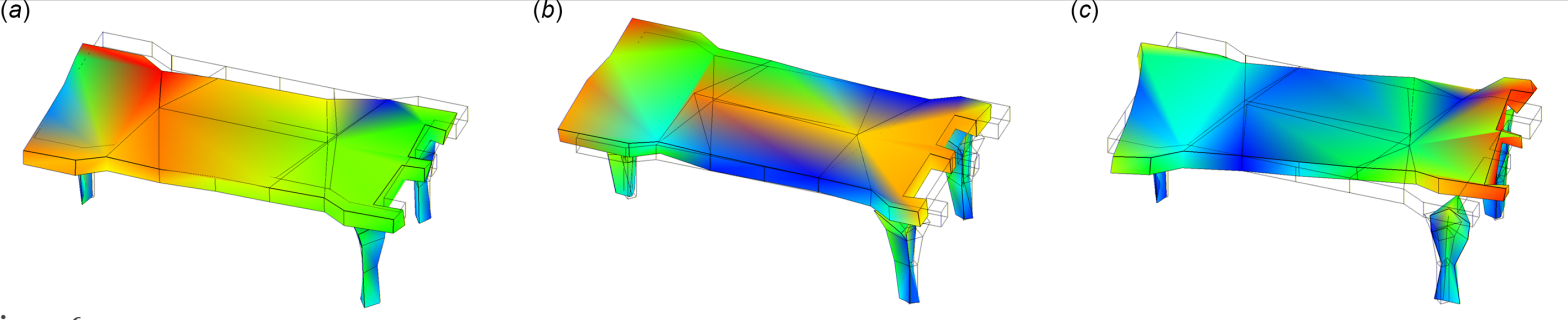
Modal shape at (*a*) 12 Hz, (*b*)18 Hz and (*c*) 25 Hz of the mirror regulating system.

**Figure 7:**
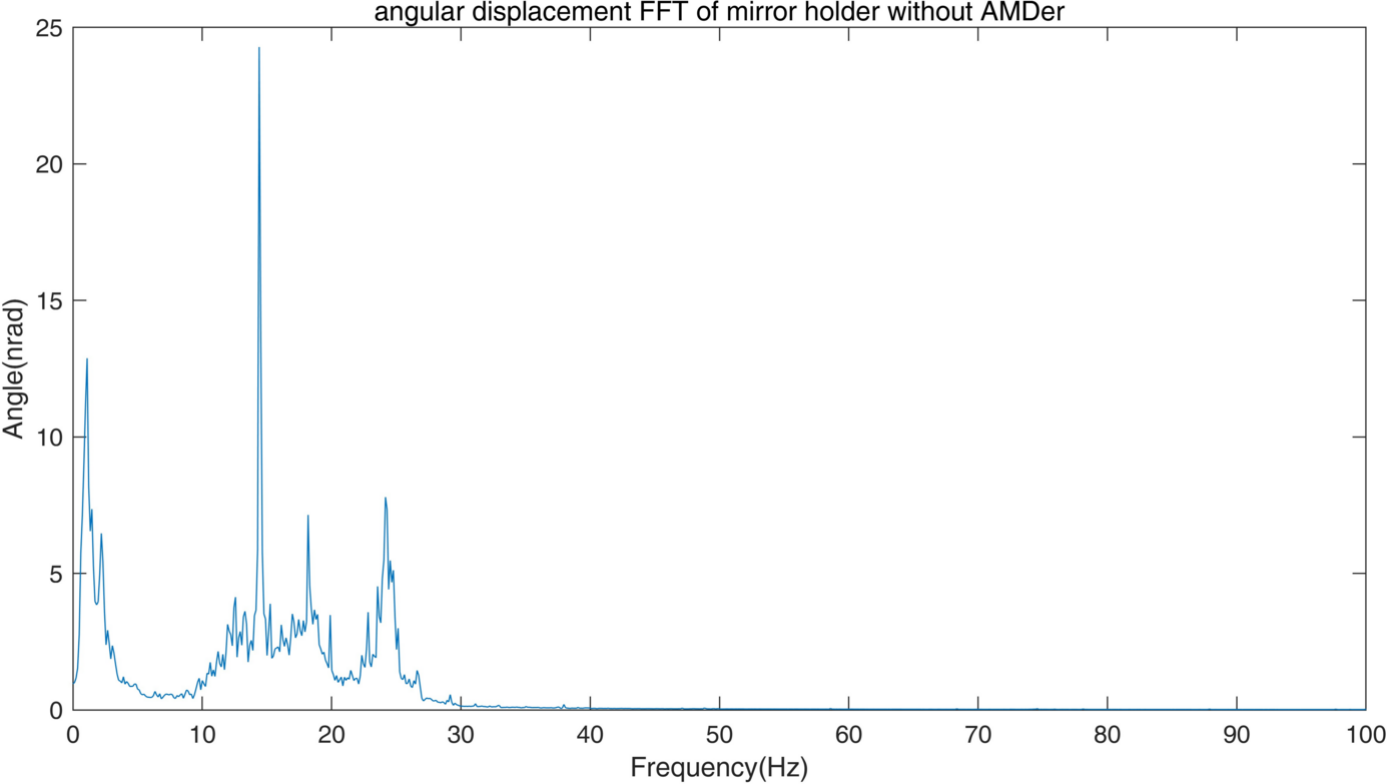
Vibration spectra of the offset mirror without the AMDer.

**Figure 8:**
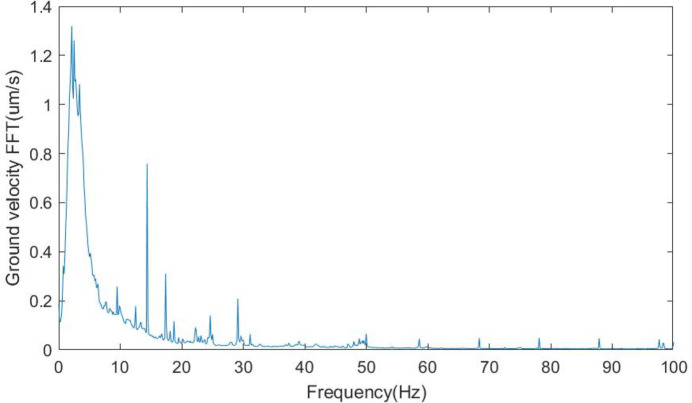
Ground vibration spectra in the vertical direction.

The modal of the system was measured with accelerometers (model 393B04, PCB), hammer (type INV9311) and *DASP* (version 11) software (http://www.coinv.com/product/59). The related pitch angular modes are shown in Fig. 6 ▸. The first pitch angular mode at 12 Hz and the third pitch angular mode at 24 Hz are due to stiffness differences on the ends of the mirror holder, the second angular mode at 18 Hz is attributed to the coupling of the translation, while the fourth mode at 160 Hz is from the fine pitch adjustment of the mirror holder. The absolute pitch angle vibration was calculated from the angular velocity by placing two velocity gauges (model 941B, produced by the Institute of Engineering Mechanics of China Earthquake Bureau) at a distance of 800 mm apart. The pitch angular spectrum is shown in Fig. 7 ▸, while the ground vibration in the vertical direction is shown in Fig. 8 ▸. The angular vibration below 5 Hz was attributed to ground excitations. The 14 Hz and 17 Hz vibrations were found to be external machine excitations from the ground, as they could be detected across the whole laboratory, and the spectra are rather narrow. The vibrations around 12 Hz, 18 Hz and 25 Hz are attributed to the structural modal vibrations of the adjustments. The fourth mode from the fine pitch adjustment was seen to be of little affect because of its high Eigen frequency.

### 3.2. Introduction of the AMDer

**Figure 9:**
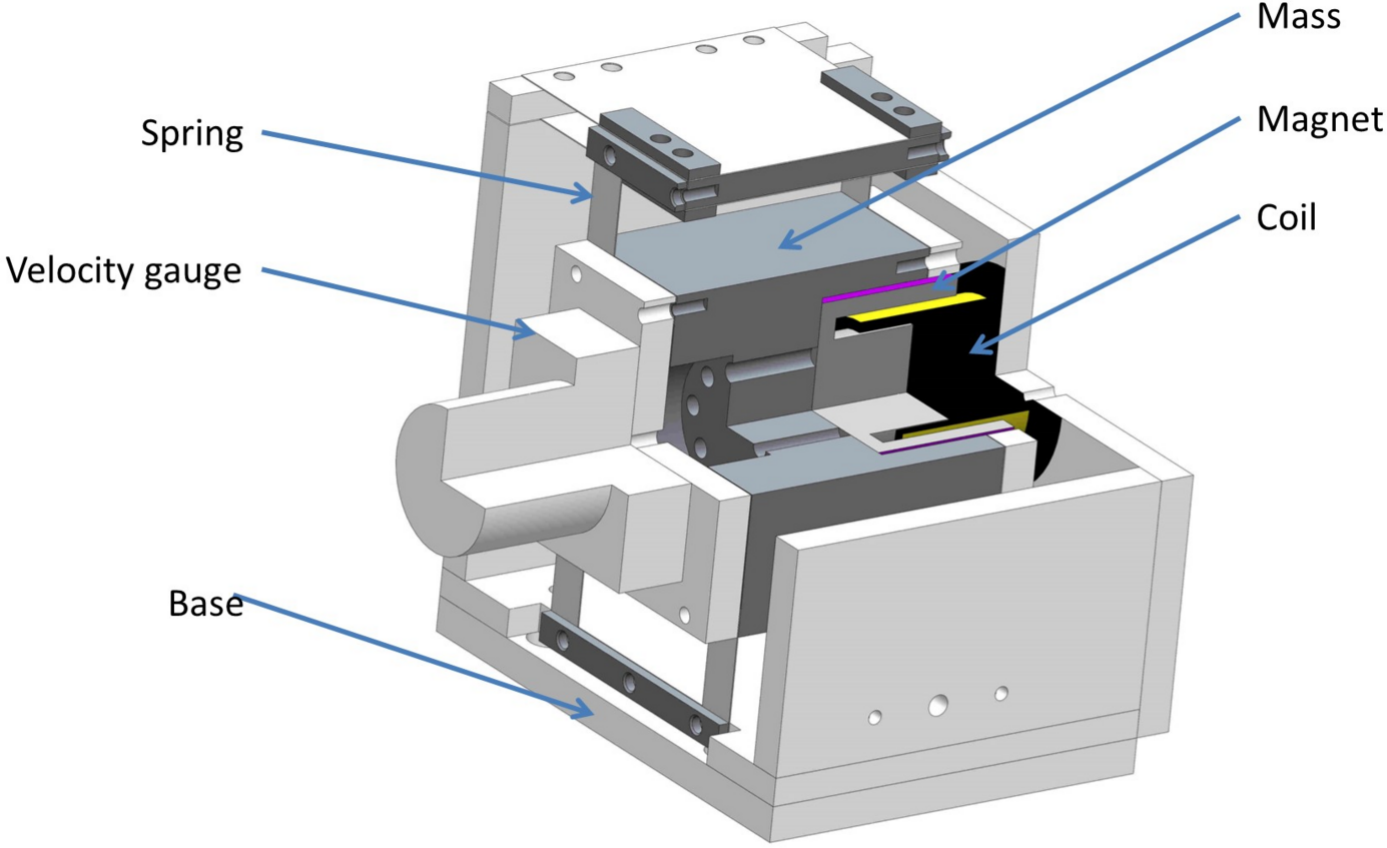
Structure of the AMDer.

**Figure 10:**
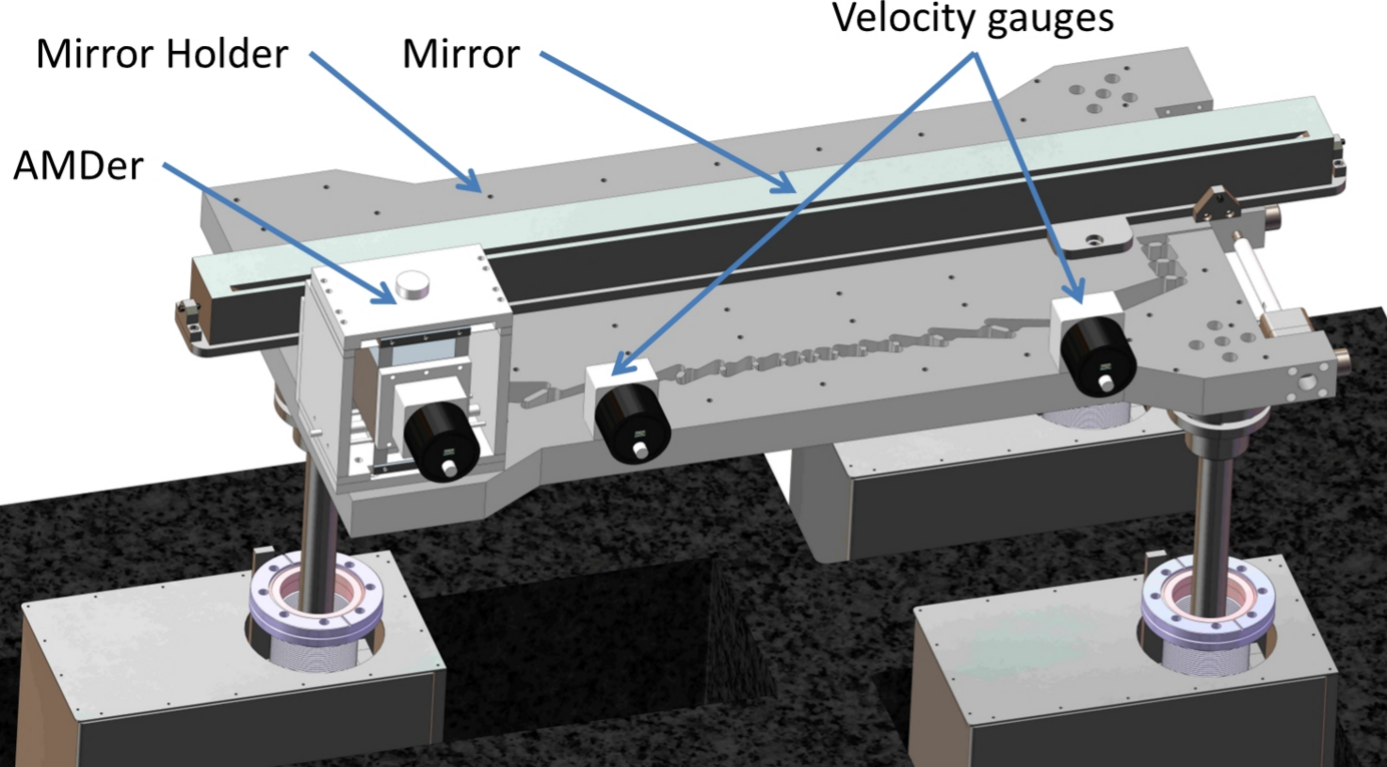
Test setup of the pitch angular vibration damping.

A 3D model of the AMDer is shown in Fig. 9 ▸; the actuator is a voice coil from Akribis (type AVM-60.25). The magnetic part is fixed to the mass, while the coil part is fixed to the base. The mass is connected to metal pieces serving as springs, and a velocity gauge (model 941B mentioned above) is also fixed to the mass for the IAVF. The signal acquisition and feedback controller is implemented in the NI cRIO-9042 RT/FPGA system: the PID controller was implemented at a 25.6 kHz sample rate. The key parameters of the AVM-60.25-type VCM and 941B-type velocity gauge are listed in Table 2 ▸. Two velocity gauges (model 941B) are fixed to the mirror holder to measure the angular velocity as
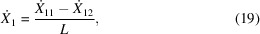
where 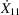 and 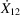 are the velocities measured by the two gauges, and *L* = 413 mm is the distance between the two gauges. The whole test setup is shown in Fig. 10 ▸. For easy mounting, the velocity gauges are fixed on the mirror holder rather than on the mirror dummy, because the pitch angular vibration on the mirror and the holder are almost the same by our measurement.

## 4. Test results and discussions

### 4.1. Characteristic rest

**Figure 11:**
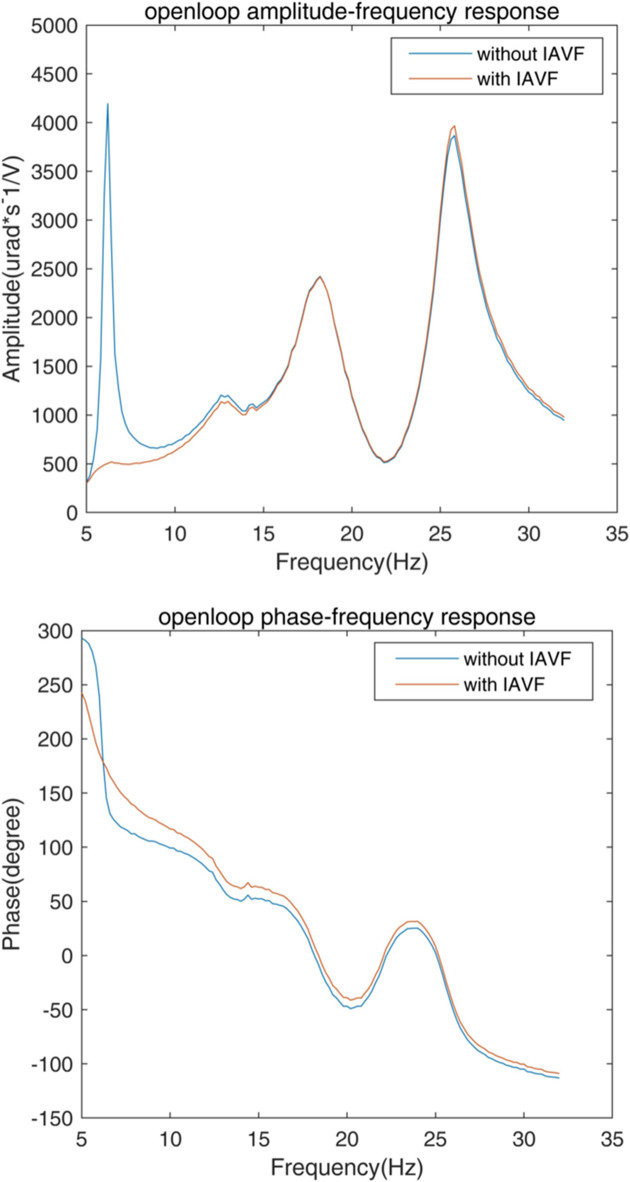
A comparison of the secondary path frequency response with and without IAVF.

Before the performance test, the frequency response characteristics *G*_OLFR_ were obtained by driving the actuator with a sinusoidal frequency scanning voltage and recording the amplitude ratio
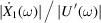
and phase difference
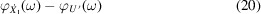
at each excitation frequency ω. As shown in Fig. 11 ▸, these resonant peaks agree well with the three modals contributing to the main vibration shown in Fig. 7 ▸; the peak at 6 Hz is caused by the AMDer.

In the absence of extra damping, the resonance of the AMD is only damped by the properties of the material; since the springs are made of stainless steel, the damping ratio was very small, so the open-loop response amplitude *G*_*s*_(*j*ω_*a*_) is too high to implement a high-gain velocity feedback, which will limit the performance of modal vibration attenuation. With IAVF (*p*_2_ = −4.58 × 10^−5^ µm s^−1^ V^−1^), *G*_*s*_(*j*ω_*a*_) decreases significantly while the change of amplitude at the natural frequency of all the controlled structure resonance *G*_*s*_(*j*ω_k_) is negligible, which indicates that the modal vibration attenuation performance is improved.

**Figure 12:**
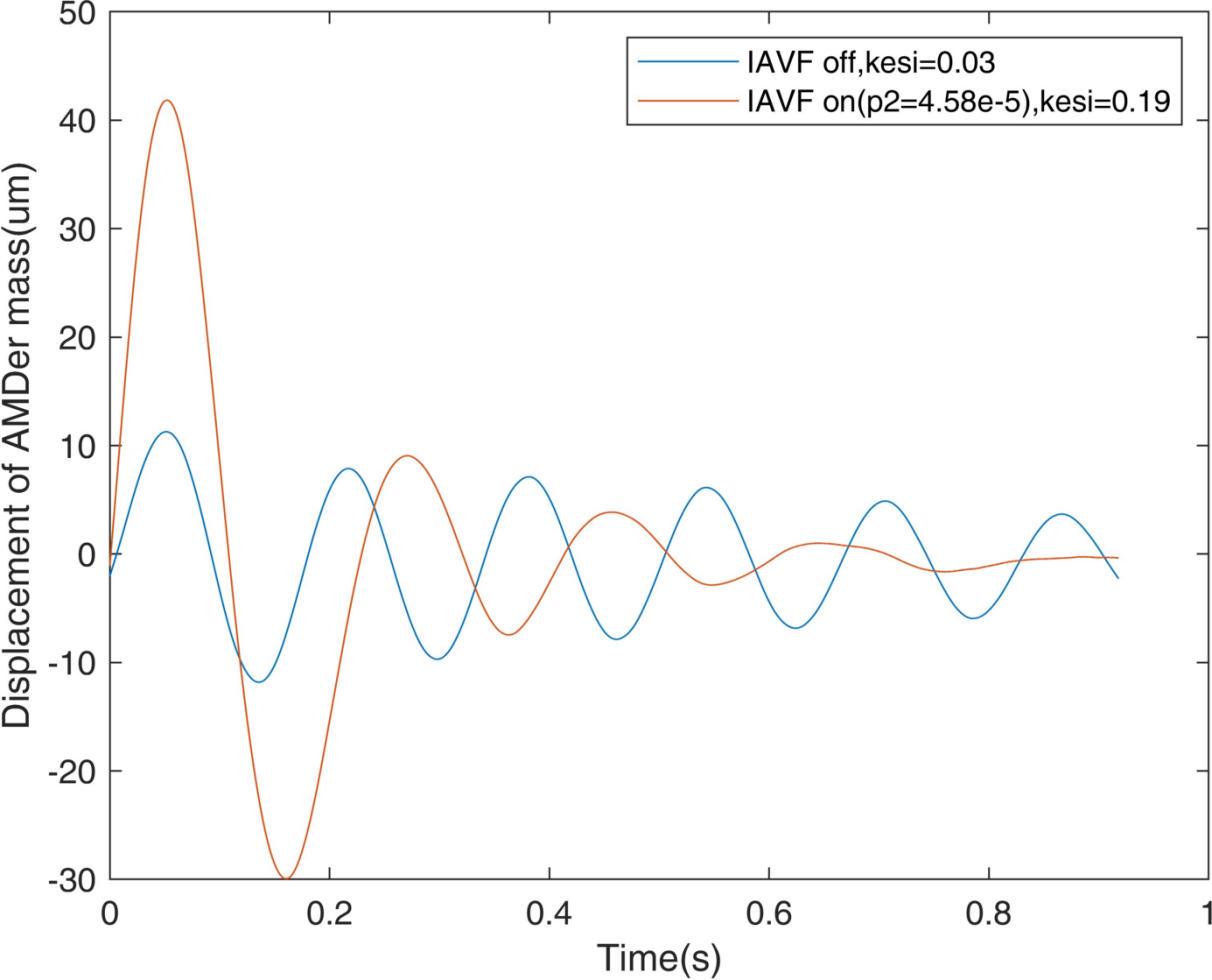
Comparison of the damping ratio with and without IAVF.

The damping ratio was also fitted according to the waveform by the impulse response of the AMDer as shown in Fig. 12 ▸. By fitting the wave with the following equation,
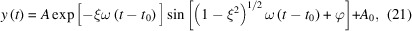
the damping rate, ξ, of the resonance of the AMDer increases from 0.03 to 0.19 with IAVF.

### 4.2. Performance test and discussions

**Figure 13:**
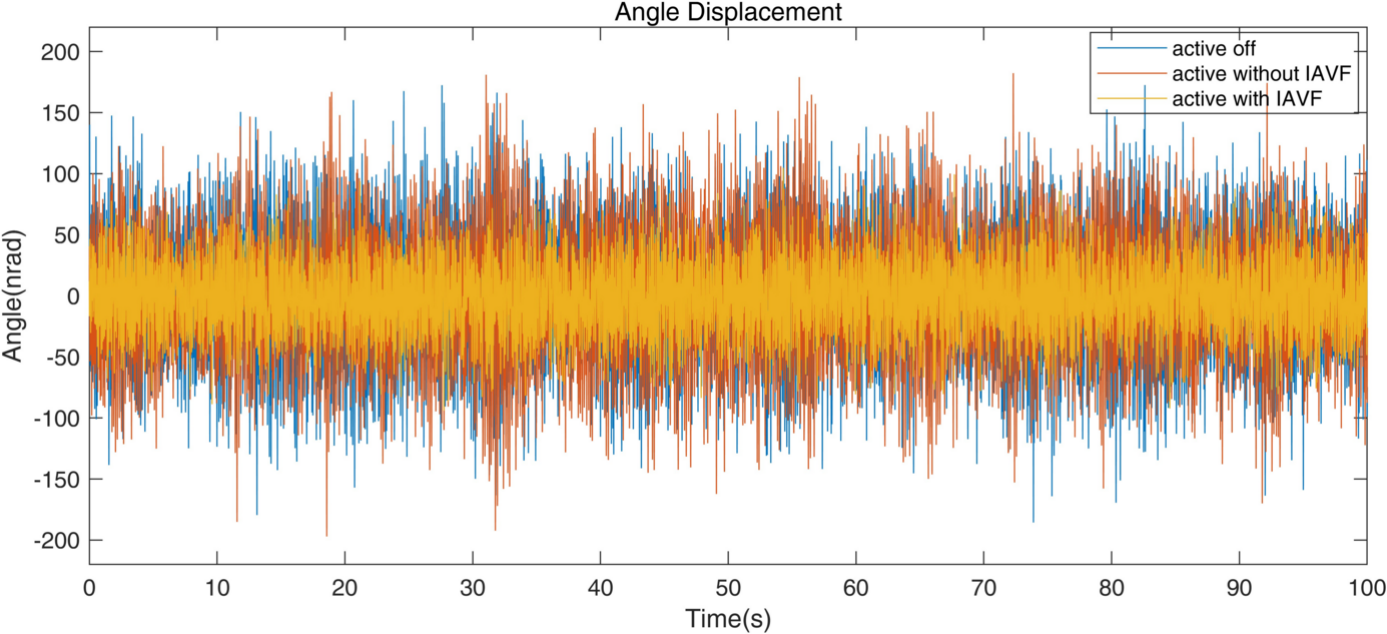
Angular vibration waveform comparison for the cases of active off, active without IAVF (*p*_1_ = 1.2 × 10^−4^ µrad s^−1^ V^−1^, *p*_2_ = 0 µm s^−1^ V^−1^) and active with IAVF (*p*_1_ = 9.3 × 10^−4^ µrad s^−1^ V^−1^, *p*_2_ = −4.58 × 10^−5^ µm s^−1^ V^−1^).

**Figure 14:**
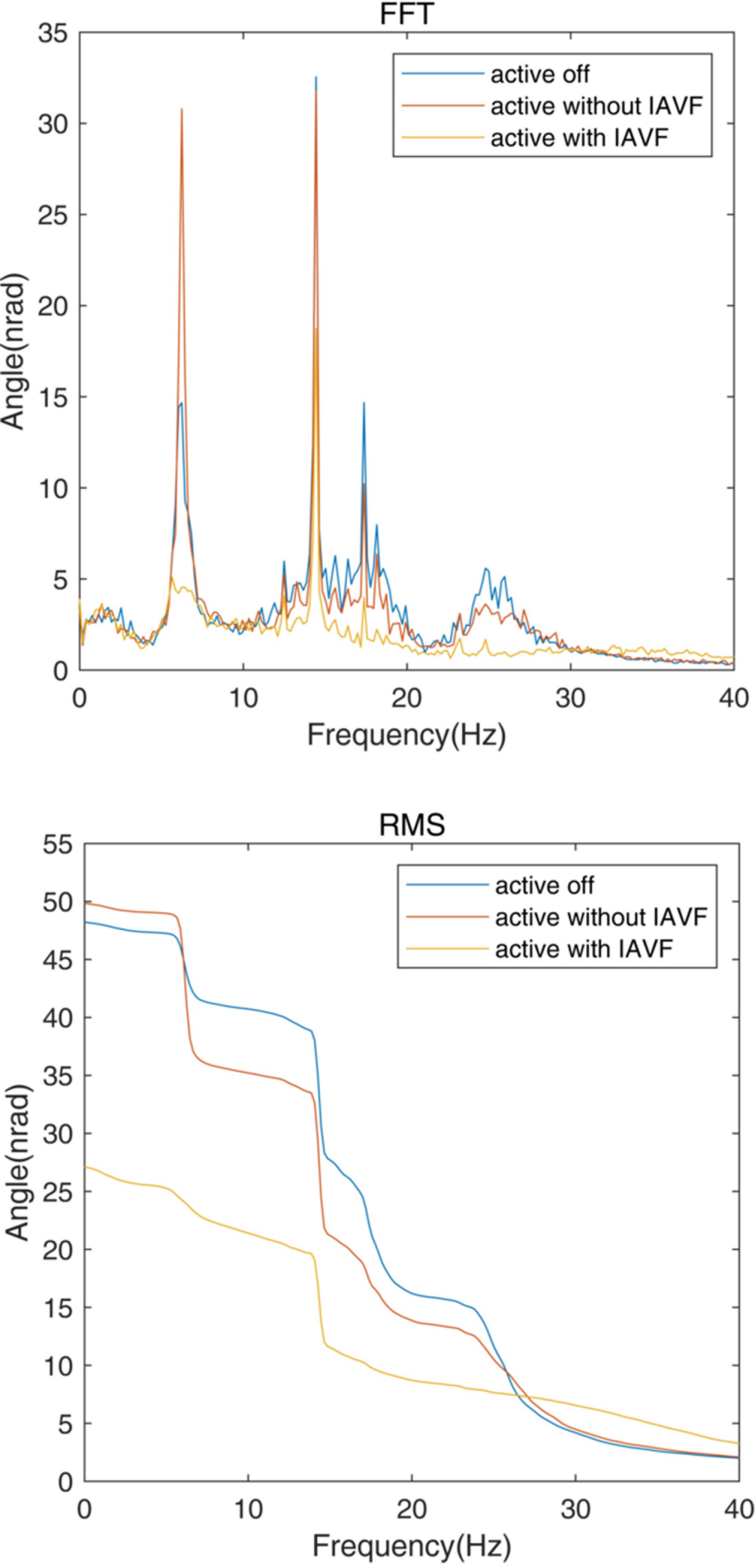
FFT (top) spectra and RMS (bottom) of the angular vibration comparison for the cases of active off, active without IAVF (*p*_1_ = 1.2 × 10^−4^ µrad s^−1^ V^−1^, *p*_2_ = 0 µm s^−1^ V^−1^) and active with IAVF (*p*_1_ = 9.3 × 10^−4^ µrad s^−1^ V^−1^, *p*_2_ = −4.58 × 10^−5^ µm s^−1^ V^−1^).

The pitch angular vibrations of the mirror holder measured by the two velocity gauges for different conditions are shown in Fig. 13 ▸. Spectra and the accumulated RMS are shown in the top and bottom panels, respectively, of Fig. 14 ▸. When the AMDer with IAVF is off, there is an additional peak at about 6 Hz compared with the spectrum in Fig. 7 ▸ without the AMDer, which is due to the natural frequency of the AMDer. Owing to the spillover effect, the damping factor with normal AMD is limited due to vibration increase at 6 Hz; the performance is then also limited. However, when the AMDer with IAVF is active, the peaks at around 6 Hz, 12 Hz, 18 Hz and 25 Hz are significantly decreased. Even at 14 Hz, the vibration caused by some external excitation is also attenuated from 26 nrad to 15 nrad. As a result, the RMS above 1 Hz of angular vibration of the mirror holder drops from 47 nrad to 27 nrad.

## 5. Conclusion

Active mass damping with IAVF was introduced to attenuate the structural angular vibration of a mirror system for FEL beamlines. Performance tests demonstrated that the IAVF can reduce the spillover effect and further improve the structural vibration attenuation performance. By comparing the angular vibrations with and without the active mass damping, the angular vibration of the mirror system was improved from 47 nrad to 27 nrad above 1 Hz in RMS.

## Figures and Tables

**Table 1:** Parameters of the AMD model

Mass of controlled structure, *m*_1_	10^−5^
Damping rate of controlled structure, ξ_1_	0.05
Natural frequency of controlled structure, ω_1_	2π × 25 Hz
Internal mass of AMDer, *m*_2_	10^−6^
Damping rate of AMDer, ξ_2_	0.04
Natural frequency of AMDer, ω_2_	2π × 5.5 Hz
Electrical time constant, τ	1.5 ms

**Table 2:** Parameters of the actuator, sensor and electronics

AVM60.25 VCM force constant, *K*_f_	17 N A^−1^
AVM60.25 VCM resistance, *R*	5.35 Ω
AVM60.25 VCM inductance, *L*	3.82 mH
−3 db bandwidth of the 914b-type velocity gauge	1–100 Hz
Input noise of the NI 9215 module	1.2 LSB (RMS), 7 LSB (p-p)
Noise of the AD 620 In-Amp	9 nV Hz^−1/2^ @ 1 kHz, 0.28 µV p-p (0.1–10 Hz)
